# A Novel Chiropteran Circovirus Genome Recovered from a Brazilian Insectivorous Bat Species

**DOI:** 10.1128/genomeA.01393-15

**Published:** 2015-11-25

**Authors:** Francisco Esmaile Sales Lima, Samuel Paulo Cibulski, Aline Gehlen Dall Bello, Fabiana Quoos Mayer, André Alberto Witt, Paulo Michel Roehe, Pedro Alves d’Azevedo

**Affiliations:** a Molecular Microbiology Laboratory, Federal University of Health Science of Porto Alegre (UFCSPA), Porto Alegre, Rio Grande do Sul, Brazil; b Department of Microbiology, Virology Laboratory, Immunology and Parasitology, Institute of Basic Health Sciences, Federal University of Rio Grande do Sul, Porto Alegre, Rio Grande do Sul, Brazil; c Fepagro Animal Health, Institute of Veterinary Research “DesidérioFinamor” (IPVDF), Eldorado do Sul, Rio Grande do Sul, Brazil; d Health Monitoring, Centro Estadual de Vigilância em Saúde, Porto Alegre, Rio Grande do Sul, Brazil

## Abstract

This report describes the identification and characterization of a novel circovirus using metagenomic approaches in respiratory fluid samples from Brazilian free-tailed bats (*Tadarida brasiliensis*). The genome and deduced protein sequences share low identity with another circovirus recovered in distantly related bats from China.

## GENOME ANNOUNCEMENT

Viruses of the *Circoviridae* family are known to infect a wide range of vertebrates. The virions consist of naked nucleocapsids of about 20 nm in diameter, with a circular single-stranded DNA (ssDNA) genome of approximately 2.0 kb (1). The rise of high-throughput sequencing approaches has led to the identification of small circular DNA genomes in fecal samples of wild mammals and in insects and environmental samples (2–4).

Bats (order *Chiroptera*) are recognized as sources of viruses that can potentially cause disease in humans and animals. During a rabies surveillance study that took place in Southern Brazil, pharyngeal swabs from 350 healthy Brazilian free-tailed bats (*Tadarida brasiliensis*) were pooled, filtered for removal of cell debris, and subjected to ultracentrifugation to concentrate the viral population. The DNA obtained was sequenced using the Illumina MiSeq system.

A total of 1,404,764 reads were generated. These sequences were assembled into 10,712 contigs using SPAdes 3.5 assembler and analyzed using BLASTn/BLASTx with the National Center for Biotechnology Information (NCBI) databases. One contig related to *Circovirus* with a circular genome of 1,767 nucleotides (nt) was assembled.

The tentatively named *Tadarida brasiliensis* circovirus 1 (TbCV-1) displays the archetypal ambisense genome organization containing two major open reading frames (ORFs) inversely arranged, responsible for encoding the replicase (Rep) and capsid (Cap) proteins, presenting 303 and 218 amino acids, respectively. The 5′ intergenic region included a potential stem-loop structure (stem-loop motif TAGTATTAC; flanked by the inverted repeat sequence GTGCCGGGG). It was evidenced in the Rep N-terminus motifs associated with rolling circle replication (FTINN, TPHLQGF) and the deoxynucleoside triphosphate (dNTP)-binding site (GSGKS), as well as the conserved 2C helicase motif in the carboxy half of Rep (WWDGY and DRYP). The overall amino acid identity between TbCV-1 and a related circovirus genome recovered from *Rhinolophus ferrumequinum* circovirus 1 (RfCV-1) bats in China shows 75.4% of identity (5). In addition, the predicted Cap and Rep protein sequences share amino acid identities of 71.1% and 85.3%, respectively, in comparison to those from RfCV-1.

In accordance with the criteria of the International Committee on Taxonomy of Viruses (ICTV), TbCV-1 could be considered a new species of the genus *Circovirus* detected in a bat species taxonomically different and geographically distant from the closest genome recovered in China. Also remarkable is the likelihood that this virus is replicating and disseminating orally, as it was found in respiratory fluids. On the other hand, it is not yet possible to extrapolate the discussions on the pathogenic role of bat circoviruses as studies related to this issue on ssDNA sequences found worldwide are still missing.

### Nucleotide sequence accession number.

The complete genome sequence of TbCV-1 has been deposited at GenBank under accession number KT783484.

## References

[1] Delwart E, Li L 2012 Rapidly expanding genetic diversity and host range of the Circoviridae viral family and other rep encoding small circular ssDNA genomes. Virus Res 164:114–121. doi:10.1016/j.virusres.2011.11.021. 22155583 PMC3289258

[2] Blinkova O, Rosario K, Li L, Kapoor A, Slikas B, Bernardin F, Breitbart M, Delwart E 2009 Frequent detection of highly diverse variants of cardiovirus, cosavirus, bocavirus, and circovirus in sewage samples collected in the United States. J Clin Microbiol 47:3507–3513. doi:10.1128/JCM.01062-09. 19794058 PMC2772610

[3] Lima FEdS, Cibulski SP, Dos Santos HF, Teixeira TF, Varela APM, Roehe PM, Delwart E, Franco AC 2015 Genomic characterization of novel circular ssDNA viruses from insectivorous bats in Southern Brazil. PLoS One 10:e0118070. doi:10.1371/journal.pone.0118070. 25688970 PMC4331541

[4] Li L, Victoria JG, Wang C, Jones M, Fellers GM, Kunz TH, Delwart E 2010 Bat guano virome: predominance of dietary viruses from insects and plants plus novel mammalian viruses. J Virol 84:6955–6965. doi:10.1128/JVI.00501-10. 20463061 PMC2898246

[5] Wu Z, Ren X, Yang L, Hu Y, Yang J, He G, Zhang J, Dong J, Sun L, Du J, Liu L, Xue Y, Wang J, Yang F, Zhang S, Jin Q 2012 Virome analysis for identification of novel mammalian viruses in bat species from Chinese provinces. J Virol 86:10999–11012. doi:10.1128/JVI.01394-12. 22855479 PMC3457178

